# Recurrent Cholangiocarcinoma Presenting as Sister Mary Joseph Nodule After Liver Transplantation

**DOI:** 10.7759/cureus.11673

**Published:** 2020-11-24

**Authors:** Vijayadershan Muppidi, Sreenath Meegada, James D Eason, Satheesh P Nair, Rajanshu Verma

**Affiliations:** 1 Internal Medicine, Indiana University Health, Indianapolis, USA; 2 Internal Medicine, University of Texas Health Science Center, Christus Good Shepherd Medical Center, Longview, USA; 3 Medicine, University of Tennessee Health Science Center, Methodist University Hospital Transplant Institute, Memphis, USA; 4 Gastroenterology, University of Tennessee Health Science Center, Memphis, USA

**Keywords:** recurrent cholangiocarcinoma, smj nodule, liver transplantation, sister mary joseph nodule

## Abstract

Hilar cholangiocarcinoma, also known as Klatskin tumor, is the most common type of cholangiocarcinoma. It usually has a lymphatic spread and is rarely associated with an umbilical nodule, also known as Sister Mary Joseph nodule. We report a case of a 53-year-old Caucasian man with hilar cholangiocarcinoma. The patient had an inoperable tumor and was referred to our center for liver transplantation. Post liver transplantation, the patient presented with a recurrence of the carcinoma in the umbilical region. The patient was found to have Sister Mary Joseph nodule. It carries a poor prognosis, and our patient succumbed to the illness in four months. Cholangiocarcinoma carries a poor prognosis. Surgical resection and liver transplantation with neoadjuvant chemoradiation are the preferred treatment strategies. Association of cholangiocarcinoma with umbilical metastasis is rare, and our patient had an even rarer presentation in the form of recurrence with umbilical nodule post-liver transplantation. We want to increase the awareness of the rare presentation, association, and recurrence of hilar cholangiocarcinoma in the form of umbilical nodule post-liver transplantation.

## Introduction

Cholangiocarcinomas comprise approximately 10-15% of hepato-biliary malignancies and about 2% of all malignancies [1]. These cancers are usually detected in their advanced stages and are associated with a poor prognosis. They are divided into three subtypes: intrahepatic, hilar (most common), and distal cholangiocarcinomas. Hilar cholangiocarcinoma (HC) is also known as Klatskin tumor. Umbilical metastasis (Sister Mary Joseph nodule) of cholangiocarcinoma is exceedingly rare, with less than a dozen reported cases in the medical literature. We describe a case of a man who underwent liver transplantation for hilar cholangiocarcinoma (HC). However, he later developed recurrence in the form of Sister Mary Joseph (SMJ) nodule. To the best of our knowledge, SMJ from HC has not been previously described in the post-transplant setting.

## Case presentation

A 53-year-old Caucasian man presented with two months of right upper quadrant pain, obstructive jaundice (dark-colored urine, clay-colored stools) and a 25-pound weight loss to the outside hospital. Past medical history was significant for cholelithiasis. Family history included melanoma in the brother, breast cancer in the mother, and cancer in the father. The patient was a non-smoker, drank alcohol occasionally, and denied any illicit drug use. Initial computed tomography (CT) scan of the abdomen showed a 1.6 x 1.6 cm wide soft tissue density at the hepatic hilum with marked dilation of intrahepatic bile ducts to the level of porta hepatis. Both right and left hepatic ducts, along with common hepatic duct, were stented with metallic stents to relieve jaundice. He also underwent a brush biopsy of common hepatic duct stricture with endoscopic retrograde cholangiopancreatography, which showed atypical cells suggestive of adenocarcinoma. Further cytopathologic investigation with fluorescent in-situ hybridization/digital image analysis confirmed the presence of cholangiocarcinoma. He was then referred to our hospital for liver transplantation for inoperable hilar cholangiocarcinoma.

A repeat CT abdomen at our hospital showed intrahepatic biliary obstruction with a suspected 3.1 x 2.0 cm Klatskin tumor. Despite the presence of metallic stents, there was a significant intrahepatic biliary ductal dilation with the right biliary stent tip being within the region of the tumor. He underwent endoscopic ultrasound, which did not show any evidence of localized adenopathy. Given the extent of narrowing, the right biliary stent could not be repositioned. As per liver transplant protocol for hilar cholangiocarcinoma, he was treated with neoadjuvant chemotherapy with gemcitabine and cisplatin for eight weeks, followed by stereotactic body radiation therapy (67.5 Gray in 15 fractions) and then oral capecitabine. He then was listed using 28 Model for End-Stage Liver Disease (MELD) exception points for liver transplant and underwent exploratory laparotomy to assess regional lymph nodes (frozen section analyzed by a pathologist) to rule out metastatic disease. Once this was confirmed, he then underwent standard criteria deceased donor liver transplantation for hilar cholangiocarcinoma along with Roux-en-Y bile duct reconstruction (choledochojejunostomy) for donor-recipient bile duct size mismatch. The patient did well postoperatively and was discharged within a week on tacrolimus and mycophenolate mofetil with satisfactory allograft function.

**Figure 1:**
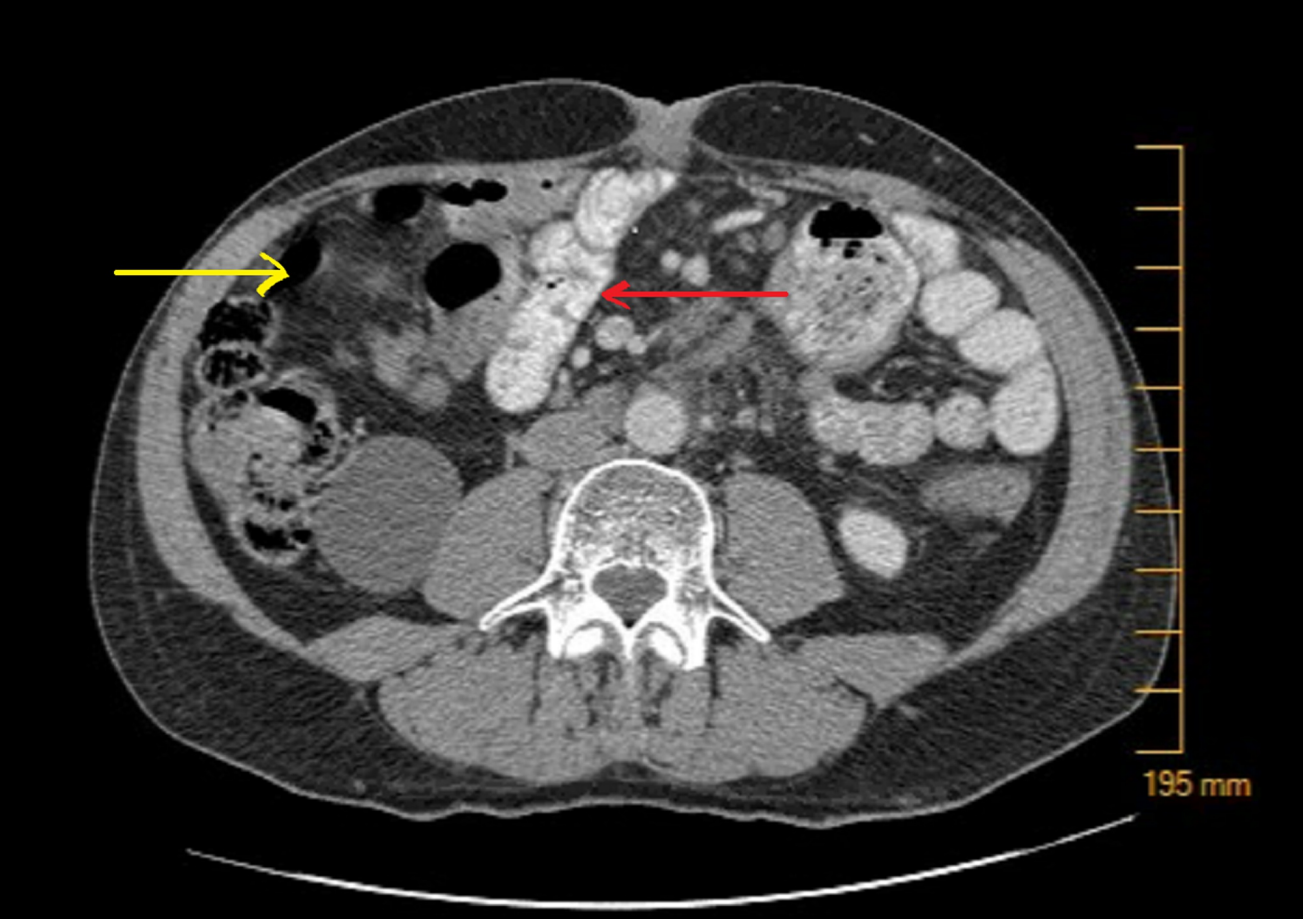
CT abdomen showed a 3.7 x 2.5 x 3.1 cm density at the root of mesentery anterior to the aorta at the level of the distal duodenum (Red Arrow), Dilated intrahepatic biliary ducts (Yellow arrow)

**Figure 2:**
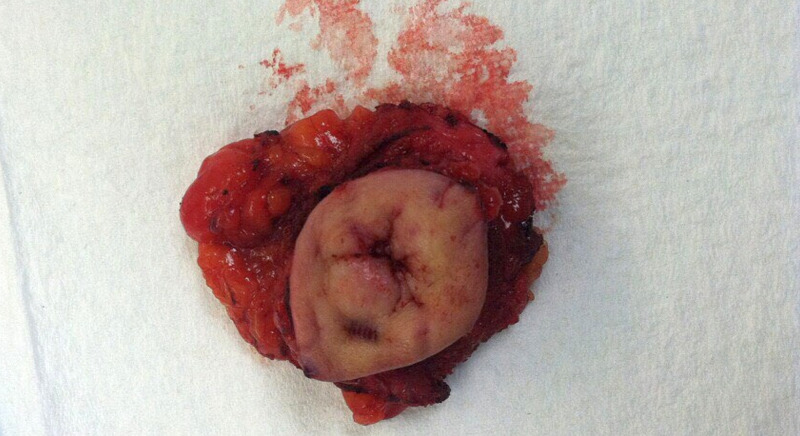
Sister Mary Joseph Nodule (Excised)

**Figure 3:**
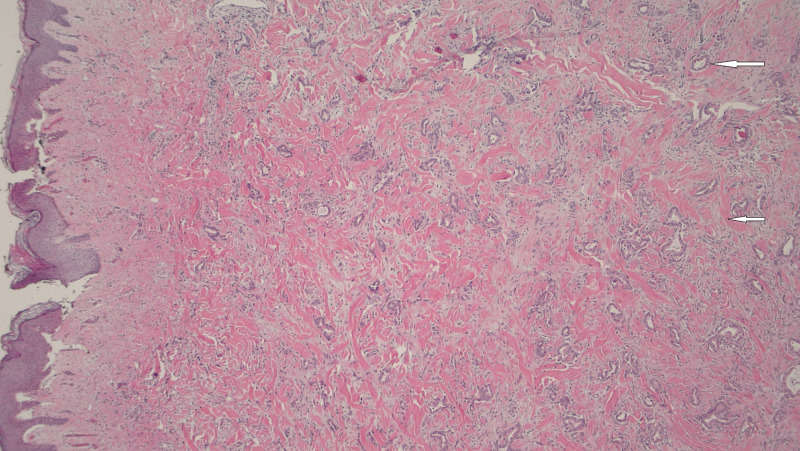
well-differentiated duct-forming cholangiocarcinoma (above arrow) with stroma (below arrow)

**Figure 4:**
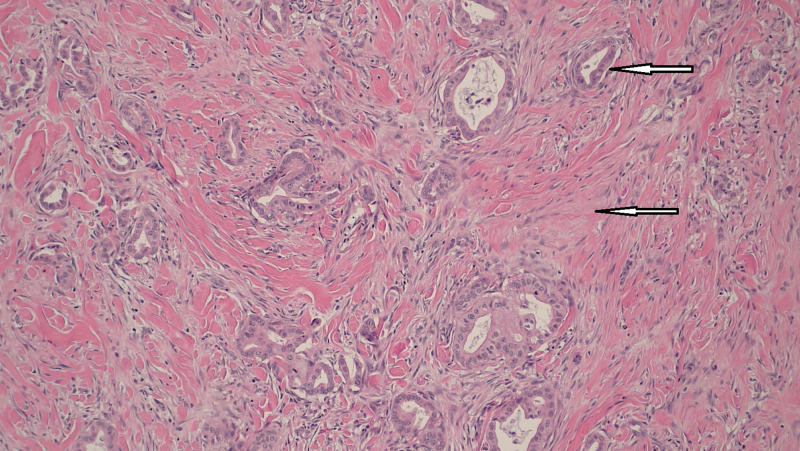
well-differentiated duct-forming cholangiocarcinoma (above arrow) and stromal tissue (below arrow)

Post-liver transplant, the patient was continued on adjuvant chemotherapy (gemcitabine and oxaliplatin) as per oncology recommendations. A year-and-a-half later, he noticed abdominal pain and abdominal fullness when a hard, tender, and erythematous umbilicus was palpated on physical exam, likely representing Sister Mary Joseph nodule. CT abdomen showed a 3.7 x 2.5 x 3.1 cm density at the root of mesentery anterior to the aorta at the level of the distal duodenum (Figure 1) with multiple shotty mesenteric lymph nodes. The patient was then taken to the operating room for the excision of the Sister Mary Joseph nodule, (Figure 2) mesenteric lymph nodes, and fascial nodule adherent to the small bowel, which required partial small bowel resection. Pathology showed well-differentiated duct-forming adenocarcinoma with abundant stroma consistent with the recurrence of the cholangiocarcinoma (Figure 3, Figure 4).

Mesenteric lymph node, as well as small bowel metastasis, showed cholangiocarcinoma. Cancer antigen 19-9 (CA19-9) rose up to 187 U/ml (normal: < 35). The patient was seen by an oncologist, and palliative chemotherapy (capecitabine) was initiated. The patient took this chemotherapy for another two months; however, he continued to deteriorate with worsening abdominal pain, weight loss, and anorexia. The patient chose home with hospice and died four weeks later at home. This was four months after his intra-abdominal (umbilical) recurrence, in the form of the SMJ nodule, was detected.

## Discussion

Hilar or perihilar cholangiocarcinoma is also known as the Klatskin tumor [2]. A majority of the cases of cholangiocarcinoma (about two-thirds) is made up of the hilar cholangiocarcinoma type. Primary sclerosing cholangitis (PSC) is a risk factor for HC and is usually diagnosed within two years of diagnosis of PSC [3]. The tumor spreads primarily through the lymphatic system. HC can spread directly into the hepatic parenchyma or hepatoduodenal ligament by direct infiltration or longitudinal spread along the bile ducts [4]. The tumor may exhibit vascular and perineural invasion. HC can also spread to the peritoneum, distant sites, and lymph nodes.

Cholangiocarcinoma carries a poor prognosis. Surgical resection was widely considered the curative option for hilar cholangiocarcinoma until liver transplantation provided an alternative strategy [5]. However, about 30 to 40% of patients with hilar cholangiocarcinoma may not have resectable tumors. The tumor is considered unresectable if it has spread to the portal vein, common hepatic artery, inferior vena cava, and contralateral hepatic lobes [6]. Sometimes, liver diseases such as primary sclerosing cholangitis may preclude surgical resection. Palliative options for hilar cholangiocarcinoma include stenting, intraluminal brachytherapy, photodynamic therapy, bypass surgery, external radiation, and systemic therapy [7]. Liver transplantation provides an alternative strategy in these hilar cholangiocarcinoma patients with irresectable and undisseminated tumors [8]. The shortage of expertise and experience has limited the availability of liver transplantation for cholangiocarcinoma to a few transplant centers. 

The survival rates post-liver transplantation in hilar cholangiocarcinoma have improved significantly. The long-term survival rates for patients undergoing liver transplantation is also higher as compared to patients treated with surgical resection. Among patients undergoing surgical resection, achieving tumor-free margins (R0) resections is the most important determinant of survival [8]. Patients with tumor-free margins have a survival rate of about 40 to 50% at five years. Among patients undergoing liver transplantation, without neoadjuvant chemoradiation, the five-year survival rates are about 50% in early stages and about 38% overall. The improvement in survival is attributed to better patient selection and adjuvant and neoadjuvant chemoradiation prior to liver transplantation as per the Mayo Clinic protocol [8]. If liver transplantation is combined with neoadjuvant chemoradiation, the survival rates may be increased to 65% at five years and 59% at 10 years [8-10].

Mean time to recurrence of HC post-liver transplantation is around 40 months with three-year and five-year recurrence rates being 5% and 12%, respectively. The most common sites of recurrence are chest wall, abdomen, bone, pancreas, and biliary tube site [11]. Umbilical nodule is a rare recurrence site for HC. Perineural invasion, lymphovascular invasion, multifocal tumors, hilar tumor, infiltrative growth pattern, and lack of adjuvant/neoadjuvant therapies are risk factors for recurrence of HC post-liver transplantation [12].

Sister Mary Joseph (SMJ) nodule is the name given to umbilical metastasis of tumors, as noticed by Sister Mary Joseph in 1928 while working with Dr. William Mayo. It is commonly associated with gastric, colonic, pancreatic, ovarian, uterine malignancies, and even lymphomas. Hematogenous spread and spread along the obliterated umbilical vein, or along falciform ligament, median umbilical ligament, and along the umbilical duct remnant are proposed modes of spread for the umbilical nodule [13]. It could be a sign of extensive metastatic disease and usually predicts a survival of 10-12 months [13].

A few cases of cholangiocarcinoma presenting as SMJ nodule have previously been described, though to the best of our knowledge, only one of them has been reported in the post-liver transplant setting [13-15].

## Conclusions

Umbilical metastasis of hilar cholangiocarcinoma is rare. This vignette emphasizes the importance of recognizing SMJ nodule as a presentation of recurrent cholangiocarcinoma in a post-liver transplant setting.
